# S100A8/A9 and sRAGE kinetic after polytrauma; an explorative observational study

**DOI:** 10.1186/s13049-017-0455-0

**Published:** 2017-11-25

**Authors:** Philippe Joly, John C. Marshall, Philippe A. Tessier, Chantal Massé, Nathalie Page, Anne Julie Frenette, François Khazoom, Soazig Le Guillan, Yves Berthiaume, Emmanuel Charbonney

**Affiliations:** 1 Faculté de Médecine, Université de Montréal, Montréal, Canada; 2 St. Michael’s Hospital and the Keenan Research Centre for Biomedical Science, University of Toronto, Toronto, Canada; 3 Axe de recherche sur les maladies infectieuses et l’immunitaires, Centre de recherche du CHU de Québec-Université Laval, and Département de microbiologie-infectiologie et immunologie, Faculté de Médecine, Université Laval, Québec, Canada; 4 Institut de recherches cliniques de Montréal, Université de Montréal, Montréal, Canada; 5 Hôpital du Sacré-Coeur de Montréal, CIUSSS-NIM, Montréal, Canada; 6 Département de médecine, Faculté de Médecine Université de Montréal, Montréal, Canada

**Keywords:** Trauma, Inflammation, Calgranulines, S100, sRAGE, Organ failure, Infection

## Abstract

**Background:**

Following tissue injury after trauma, the activation of innate immune pathways results in systemic inflammation, organ failure and an increased risk of infections. The objective of this study was to characterize the kinetics of the S100A8/S100A9 complex, a new-recognized alarmin, as well as its soluble receptor sRAGE, over time after trauma as potential early biomarkers of the risk of organ damage.

**Methods:**

We collected comprehensive data from consenting patients admitted to an ICU following severe trauma. The blood samples were taken at Day 0 (admission), Day1, 3 and 5 S100A8/A9 and sRAGE were measured by ELISA. Biomarkers levels were reported as median (IQR).

**Results:**

Thirty-eight patients sustaining in majority a blunt trauma (89%) with a median ISS of 39 were included. In this cohort, the S100A8/A9 complex increased significantly over time (*p* = 0.001), but its levels increment over time (D0 to D5) was significantly smaller in patients developing infection (7.6 vs 40.1 mcg/mL, *p* = 0.011). The circulating level of sRAGE circulating levels decreased over time (*p* < 0.0001) and was higher in patients who remained in shock on day 3 (550 vs 918 pg/mL; *p* = 0.02) or 5 (498 vs 644 pg/mL; *p* = 0.045). Admission sRAGE levels were significantly higher in non-survivors (1694 vs 745 pg/mL; *p* = 0.015) and was higher in patients developing renal failure (1143 vs 696 pg/mL, p = 0.011).

**Discussion:**

Our findings reveal an interesting association between the biomarker S100A8/9 least increase over time and the presence of infectious complication after trauma. We describe that the sRAGE decline over time is in relation with shock and markers of ischemic injury. We also confirm the association of sRAGE levels measured at admission with mortality and the development of renal failure.

**Conclusions:**

This work illustrates the importance of following the circulating level of biomarker overtime. The utilization of S1008/9 as a tool to stratify infection risk and trigger early interventions need to be validated prospectively.

## Background

Traumatic injury is the leading global cause of mortality for individuals between 15 and 44 years of age [1, 2]. Patients surviving their initial resuscitation following severe trauma are at risk of subsequent complications such as sepsis and multiple organ dysfunction syndrome (MOD), which are responsible for late morbidity and mortality [3–5].

Tissue injury secondary to trauma results in the release of endogenous molecules, that have been called damage-associated molecular patterns (DAMPs). These endogenous molecules, also known as alarmins, are released passively from necrotic cells, or actively by alternative secretion pathways, and contribute to tissue damage [6, 7].

Alarmins activate immune responses and stimulate the migration of immune cells to the site of injury (reviewed in [8]). They bind to several receptors, including the receptor for advanced glycation end-products (RAGE), Toll-like receptor 4, and the formyl peptide receptors [9].

The most extensively characterized alarmin released in human trauma is the high mobility group box nuclear protein 1 (HMGB1) which is a trigger of organ dysfunction after tissue injury [10, 11]. The release of other alarmins, such as DNA (cfDNA) and mitochondrial DNA has also been documented following trauma [12, 13].

RAGE, a pattern-recognition receptor triggering non-infectious pro-inflammatory processes in response to danger is the target of various DAMPS, including advanced glycation endproducts, members of the S100/calgranulin family, HMGB1 or amyloid-β peptide [14–16]. Its basal expression is the highest in the lung and it is a known marker of lung injury [16–18] RAGE can be cleaved by proteolysis, resulting in the circulation of its soluble component. Soluble RAGE (sRAGE) acts as a decoy receptor, which can be rapidly released after various diseases or surgeries and has been implicated in pro-inflammatory or protective phenomenon, a role that remains controversial or disease dependent [19, 20]. *Cohen* et al. measured sRAGE level in the emergency room (ER) very early after trauma (< 24 h) and showed an association with the severity of injury, coagulopathy and endothelial activation, as well as with late organ dysfunction, particularly in the kidney [21].

One other interesting well-characterized ligand of RAGE is S100A8/A9, also known as calprotectin, but not investigated in the condition of trauma. S100A8/A9 is a dimer of two small calcium-binding proteins, S100A8 and S100A9. It is highly expressed by neutrophils and monocytes, and by activated epithelial and endothelial cells [22, 23]. High persistent levels of S100A8/A9 have been documented in acute and chronic inflammation, tissue remodeling, and infection [24–27]*.* S100A8 and S100A9 regulate inflammatory responses, while S100A8/A9 is involved in bacterial clearance and inhibits microbial growth by chelating divalent cations [28, 29].

Our focused interest for the S100A8/A9 complexes derived from the context of known sterile inflammation, tissue repair and infectious risks within the trauma population. Besides, the knowledge that S100A8/A9 can amplify the endotoxin-triggered inflammatory responses of phagocytes [30], coupled to our observation of endotoxin circulation within the same cohort [31], made it attractive to explore.

The objective of this study was to characterize the kinetics of the S100A8/S100A9 complex, as well as its soluble receptor sRAGE, over time after trauma, as potential early biomarkers of subsequent organ dysfunction.

## Methods

### Population

Patients were recruited from the intensive care unit of St. Michael’s Hospital, a level I trauma center, from a study aiming at endotoxin and biomarkers measurement in severely traumatized patients [31].

Inclusion criteria were serious traumatic injury defined as an Injury Severity Score (ISS) ≥ 16 and admission in the ICU within 24 h of trauma. Exclusion criteria applied to the source cohort were the age ≤ 18 years and other reasons related to the interference with the endotoxin test, like the diagnosis of infection on admission, Von Willebrand’s disease or the need for plasma exchange previous to admission.

### Clinical data and outcomes

Comprehensive clinical data were collected, including baseline demographic characteristics, Injury Severity Score (ISS), type of injury (blunt vs penetrating), vital signs (Blood Pressure, Temperature), physiological data (admission lactate and base deficit) in the emergency room (ER), resuscitation information (transfusion, fluid administered during the first 24 h, use of vasopressors), surgical interventions during the first 48 h. Multiple Organ Dysfunction (MOD) scores, were calculated daily over the first 10 days; scores were summed to calculate cumulative MODS over 10 days. The worst score per organ system within the 10 days was reported. Duration of stay, mechanical ventilation and mortality were recorded. Shock beyond the admission day was defined as the need for vasopressors.

A formal adjudication process was used to establish a diagnosis of nosocomial infection. The committee reported the occurrence of infection based on comprehensive clinical data and all bacteriological data; disagreements were resolved by consensus. The day of infection was determined as the bacteriological sampling day or antibiotic start.

### Samples collection and measurements

Plasma samples were available in 38 patients of the initial cohort (*N* = 48); the other samples were either insufficient, lost or already used for laboratory expermiments. They were collected within 24 h after trauma (Day 0) and on Days 1, 3 and 5. A sample of 3 ml of blood was collected in an EDTA tube, was centrifuged and the plasma was aliquoted and stored at −80 °C. The samples were analyzed after completion of the study. sRAGE was measured by ELISA (Enzo Biochem Inc., Farmingdale, NY, USA). S100A8/A9 was measure by an ELISA, previously described from Dr. Tessier’s lab, with a detection limit of 100 pg/mL and high specificity [32].

### Statistical analysis

Continuous variables were reported using median (IQR) or means (SD) accordingly. Categorical variables were reported as proportions. Simple comparison were done using Mann-Whitney test. Serial samples were pair analyzed using Wilcoxon signed-rank test or Friedman’s two-way analysis of variance by rank for the four time points, including complete data points. Correlations were assessed by Spearman correlation test.

## Results

The demographic characteristics of the study population are presented in Table 1. Blood samples were available for analysis in 38 (100%) patients on day 0, in 36 (95%) on day 1, in 33 (87%) on day 3, and in all four sampling time points for 30 (79%) of the enrolled subjects.

**Table 1:** Characteristics of Patients

Characteristics	*N* = 38
Age (years), median (IQR)	46.5 (23–58.5)
Gender (men/women), *n* (%)	27 (71) / 11 (29)
ISS^a^, median (IQR)	39.5 (30; 50)
Blunt Trauma, *n* (%)	34 (89.5)
Base deficit^b^ > 6 mmol/L, *n* (%)	28 (73.7)
First lactate (mmol/L), mean (SD)	3.5 (1.9)
Shock, *n* (%)	26 (68.4)
Vasopressors on Day 0, *n* (%)	11 (28.9)
PRBC ^c^ in 24 h, median (IQR)	2.5 (0; 8)
Fluid administer (L) in 24 h, median (IQR)	7.9 (5.1; 11.9)

^a^Injury Severity Score; ^b^ In absolute value; ^c^ Number of Packed Red Blood Cell

The median duration of ventilation was 10 (IQR: 6–16) days, ICU length of stay (LOS) was 9 (IQR: 4–13) days and hospital LOS was 20 (IQR:11–35) days. The mortality was 21%, with 3 patients out of 8, who died within the sampling time (before day 5). An infection was adjudicated to be present in 13 (34%) of patients during the 10 days of observation; the median day of infection was Day 4 (range: 2–9).

**Fig. 1:**
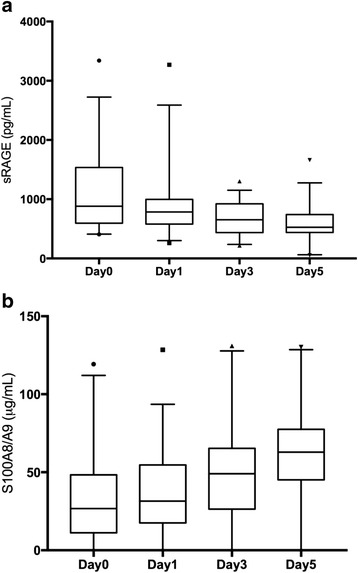
Time course of sRAGE and S100A8/9 expression over time

The S100A8/A9 complex increased significantly over time (*p* = 0.001; Friedman test), whereas the sRAGE circulation levels decreased significantly (*p* < 0.0001; Friedman test), as shown in Fig. 1.

### S100A8/9 clinical associations

The S100A8/9 complex was under the detection threshold in 10 out of the 137 samples tested. S100A8/9 complex levels showed no association with mortality. In patients who were the more severely injured with an ISS > 35 at admission, the level of S100A8/9 was significantly lower at Day 5 (Median, 52.7 vs 70.3 mcg/mL, *p* = 0.020; Mann-Whitney) as well as for the maximum reached (*p* = 0.038).

**Fig. 2:**
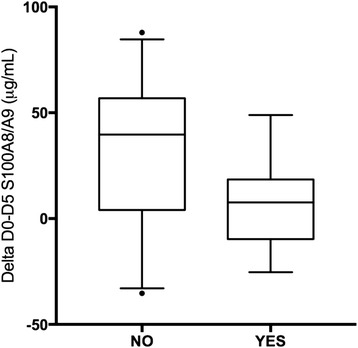
Infection and increase of S100A8/9 over time (D0-D5)

The increase in the circulating level of the complex S100A8/9 over time (Delta from Day 0 to Day 5 value; Fig. 2) was significantly smaller in patients developing infection (Fig. 2; Median, 7.6 vs 40.1 mcg/mL, *p* = 0.011; Mann-Whitney); baseline values (Day 0) were not different for those developing an infection from the other patients (Median, 20.9 vs 27.7 mcg/mL, *p* = 0.79). No association with MODS was found. Finally, since sRAGE is a decoy receptor to S100 proteins, inversed correlation of delta over time was tested, with none found.

### sRAGE clinical associations

Although levels were comparable on admission, sRAGE levels showed greater decline in patients still in shock at day 3 (918 vs 550 pg/mL; p = 0.020; Mann-Whitney) and 5 (644 vs 498 pg/mL, *p* = 0.045; Mann-Whitney). The first lactate level correlated with sRAGE decline over 5 days (rho = 0.48, *p* = 0.012); further, sRAGE levels on day 3 (rho = −0.42; p = 0,027) and 5 (rho = −0,46; p = 0,019) were inversely correlated with the amount of blood products transfused during the first 24 h. No association was found with ISS or the fluid requirement within the first 24 h.

Finally, the sRAGE levels on day 0 and day 1 were significantly higher in the non-survivor (Table 2). The sRAGE levels on day 0 correlated with the maximal renal MODS score (rho = 0.48, *p* = 0.005). Day 0 sRAGE levels were significantly higher (1143 vs 696 pg/mL, *p* = 0.011) in the 16 patients who developed renal dysfunction. (*N* = 16) than in those who did not (*N* = 21).

**Table 2:** Admission sRAGE levels and mortality

	Non-survivors	Survivors	p*
sRAGE Day 0, pg/mL	[*N* = 8] 1694 (749;2324)	[*N* = 30] 745 (409;1210)	0.015
sRAGE Day 1, pg/mL	[*N* = 7] 925 (614;2023)	[*N* = 29] 760 (284;939)	0.048

Values are represented as median (min;max). *Mann-Whitney

When non-survivors were excluded, the day 3 sRAGE level was inversely correlated with the ventilation duration (rho = − 0.39, *p* = 0.043).

## Discussion

Our study is showing that S100A8/9 levels increased over time, but is lower by Day 5 in patients with the highest ISS at admission. Even more intriguing, our findings reveal an association with infectious complication in patients with the least increase of S100A8/9 circulation over time.

We also complete the data of *Cohen* et al. [21] and show that the sRAGE levels persist following multiple trauma. We do not find any association with ISS or thoracic injury, but report that its decline over time is in relation with shock and markers of ischemic injury (lactate, need for transfusion).

We also confirm the observation regarding the association of sRAGE at admission and the development of renal failure. Finally, we find significantly higher sRAGE levels on day 0 and day 1 in non-survivors. Our small cohort does not allow us to adjust for other characteristics.

*Cohen* et al. reported that sRAGE plasma levels, measured at one time-point, increased early after trauma admission in the ER and correlated with the severity of traumatic injury and organ dysfunction particularly renal [1, 2, 21]. We describe a declining sRAGE kinetic after trauma, which question the reason of this phenomenon. It is in contradiction with the theory of renal accumulation suggested by Cohen, but reflects probably the initial insult that will lead to further deterioration of the organ function [21].

The alternative explanations of “shedding” exhaustion versus the consumption of this decoy receptor could be hypothesized, but cannot be elucidated with our work. Indeed, a variety of alarmins have the potential to target a circulating receptor, including HMGB1, which has been showed by *Peltz* et al. to be persistently elevated up to 136 h after trauma [32]. The further evaluation of this phenomenon would be of interest.

Since, we could assume that the principal source of sRAGE come from the lung where its expression is the highest by far [18], the association with respiratory failure would make sense. We find no association with respiratory failure (MODS or P/F ratio). However, an inverse correlation of sRAGE at day 3 with the ventilation duration could be the reflect of higher alarmin’s release, if we adhere to the sRAGE consumption theory.

The S100A8/9 circulating complex that we studied is also called calprotectin and actively secreted during phagocytosis. It is considered an endogenous DAMP and activator of TLR4 [33]. It is involved in inflammation, enhancement of neutrophils phagocytosis and bacterial clearance [29, 34]. In the setting of trauma cellular injury and ischemia, we were not surprised to measure an increasing level of circulation. Interestingly, the more severely injured reached lower maximum circulating levels, particularly after 5 days.

The patients where the S100A8/9 circulating complex did not increase significantly between D0 and D5 were more prone to infection. Could this be an explanation for the higher risk of infection in severe trauma [3–5, 35]? It could be speculated that the lack of increase of S100A8/9 over time could be the indirect sign of immune cells exhaustion and decreased immune defense against infections. However, the persistence of systemic inflammation renders the prediction of infection challenging after trauma, as illustrated for example with the study of C-reactive protein [36]. Further studies are needed to validate the role of S100A8/A9 as a biomarker to predict infection after trauma and test its utility for antibiotic early prescription.

As the literature reports that S100A amplify the endotoxin-induced inflammation, we have tested their association (data not shown) [30]. We found none, probably due to our small number of patients and other cofounders.

This study has some limitations. First, it is a relatively small cohort of patients to study the potential of molecular biomarkers to predict outcome post trauma and prognostic inferences are limited by our univariate comparisons; a larger cohort is needed in order to confirm the predicted value of our biomarkers in a multivariate model. Second, the physiological evaluation was limited to general parameters and not determined by more targeted measurement. Finally, although the infectious outcome was not protocolized, it was nevertheless established by adjudication.

## Conclusions

In summary, our study is the first to describe the circulation of the S100A8/9 complex in an acute setting and shows its association with infectious risks. We also report for the first time the kinetics of sRAGE in the early phase after multiple trauma. This work illustrates the importance of following the circulating level of biomarker overtime after trauma in order to better define their potential to predict post trauma complications. The utilization of S1008/9 as a tool to stratify infection risk and trigger early interventions need to be validated prospectively.
